# Choleraic Diarrhœa

**Published:** 1873-08

**Authors:** E. Mason

**Affiliations:** Wetumpka, Ala.


### CHOLERAIC DIARRHŒA.

WETUMPKA, ALA., July 30, 1873.

*Editors of the Atlanta Medical and Surgical Journal:*

The appearance of cholera at various places throughout the country, and the general prevalence of bowel affections, induce me to offer the following brief experience in cholera morbus, or what might be better termed choleraic diarrhœa, which has prevailed quite extensively in this locality during the past two months.

The attacks are usually sudden, without premonitory warning, the prominent symptoms being nausea and vomiting, excessive purging, and intense pains in the stomach and bowels; pulse quick and small; skin generally too cold; thirst very great; countenance anxious, indicating much suffering; the evacuations from the bowels large and watery—muddy or milky appearance would better describe them—very offensive.

The following treatment, with slight modifications to suit age and special indications, has proven entirely successful: Apply immediately, when called to a case, a large sinapism over the stomach and bowels, and if there is a tendency to collapse, also to the extremities and spine. Give a teaspoonful (to an adult) of the following mixture every half hour or hour, diluted in a small quantity of cold water, until the urgent symptoms are checked:

℞—Tinct. Opii., Tinct. Camph., Ess. Zingib., Ess. Pip'menth. aa ℥ ss. Misce.

To relieve thirst, give small pieces of ice occasionally. As soon as the violence of the disease is arrested, give one of the following pills every four hours, until they act properly on the suspended secretions, or until four are given; then, if necessary, give a small dose of castor oil and spirits of turpentine:

℞—Hydrag. Chlorid. Mit. gr. xvi.
Bismuth subnit. gr. xx.
Morphiæ Acet. gr. i.

Misce et ft. pill No. 4.

Where the pain and cramp are severe and stomach irritable, give a full dose of morphine hypodermically.

The remedies here suggested are neither new nor original, but the combinations have proven so efficient in my hands, for this form of disease, they are submitted with the hope, if deemed worthy, they may find a place in your valuable JOURNAL.

E. MASON, M.D.

